# Tailored chemotherapy: Innovative deep‐learning model customizing chemotherapy for high‐grade serous ovarian carcinoma

**DOI:** 10.1002/ctm2.1774

**Published:** 2024-09-07

**Authors:** Se Ik Kim, Sangick Park, Eunyong Ahn, Jeunhui Kim, HyunA Jo, Juwon Lee, Untack Cho, Maria Lee, Cheol Lee, Danny N. Dhanasekaran, Taejin Ahn, Yong Sang Song

**Affiliations:** ^1^ Department of Obstetrics and Gynecology Seoul National University College of Medicine Seoul South Korea; ^2^ Department of Advanced Green Energy and Environment Handong Global University Pohang South Korea; ^3^ Research Group Foretell My Health Pohang South Korea; ^4^ Cancer Research Institute Seoul National University College of Medicine Seoul South Korea; ^5^ Department of Agricultural Biotechnology WCU Biomodulation Seoul National University Seoul South Korea; ^6^ Interdisciplinary Program in Cancer Biology Seoul National University College of Medicine Seoul South Korea; ^7^ Department of Pathology Seoul National University College of Medicine Seoul South Korea; ^8^ Stephenson Cancer Center University of Oklahoma Health Sciences Center Oklahoma City Oklahoma USA; ^9^ Department of Life Science Handong Global University Pohang South Korea; ^10^ Department of Obstetrics and Gynaecology Myongji Hospital Hanyang University College of Medicine Goyang South Korea

Dear Editor,

The study presents a novel RNA‐seq‐based deep‐learning model for predicting the chemoresistance of platinum‐based therapy in high‐grade serous ovarian carcinoma (HGSOC), aiming to personalize chemotherapy and improve patient outcomes. By leveraging diverse transcriptome datasets of ovarian tissue and employing deep ensemble learning techniques, the model prioritized to predict chemo‐resistant HGSOC patients after initial platinum‐based chemotherapy with high performance prioritized to sensitivity (sensitivity 100%, specificity 54.1% and area under the curve [AUC] 0.85). This may offer treatment strategies and enhance clinical reliability.

HGSOC remains a significant health burden with high mortality rates worldwide, often diagnosed late due to ineffective screening.^1^ Furthermore, despite extensive surgery and chemotherapy, chemo‐resistance remains a major challenge of platinum‐based therapy in HGSOC, necessitating accurate prediction methods to improve patient outcomes and guide treatment decisions. Predicting the chemo‐sensitivity of platinum‐based therapy is the very first step of the personalized medicine for HGSOC, as it may offer incorporation of targeted agents.^2^ Genetic profiles offer potential in predicting resistance of platinum‐based chemotherapy in HGSOC, supplementing clinicopathologic data inadequacies.^3^ Yet, reliance solely on genomic data faces challenges due to tumour heterogeneity.^4^ However, epigenetic factors, and DNA methylation patterns, offer promise in chemotherapy response prediction, while RNA‐seq data aids in chemo‐resistance prediction, requiring further validation for the clinical applicability of a small number of samples.^5^ Gene expression difference among racial groups in HGSOC is also confounding for accurate prediction of survival outcome.^6^

Here, we adopt strategical approaches to extract universal chemo‐resistance traits from public data with diverse ethnic backgrounds aiming for prediction accuracy in a small sample size. We utilized RNA‐seq of fresh‐frozen primary ovarian cancer tissue from The Cancer Genome Atlas (TCGA), Seoul National University (SNUH) and Patch et al.’s dataset (Patch).^7^ TCGA includes a majority of Caucasians, comprising 208 (chemo‐resistant group: 149, chemo‐sensitive group: 59) HGSOC patients. Patch comprises 40 (24, 16) Australian HGSOC patients. SNUH included 86 (14, 72) Korean HGSOC patients, who applied the same resistance criteria (no recurrence within 6 months) after initial platinum‐based chemotherapy. No significant differences were observed in age, CA‐125 levels, or FIGO stage between chemo‐resistant and chemo‐sensitive cases (Table S1).

The study proceeded through three phases: data preprocessing, gene selection, and deep learning (Figure 1).

**FIGURE 1:**
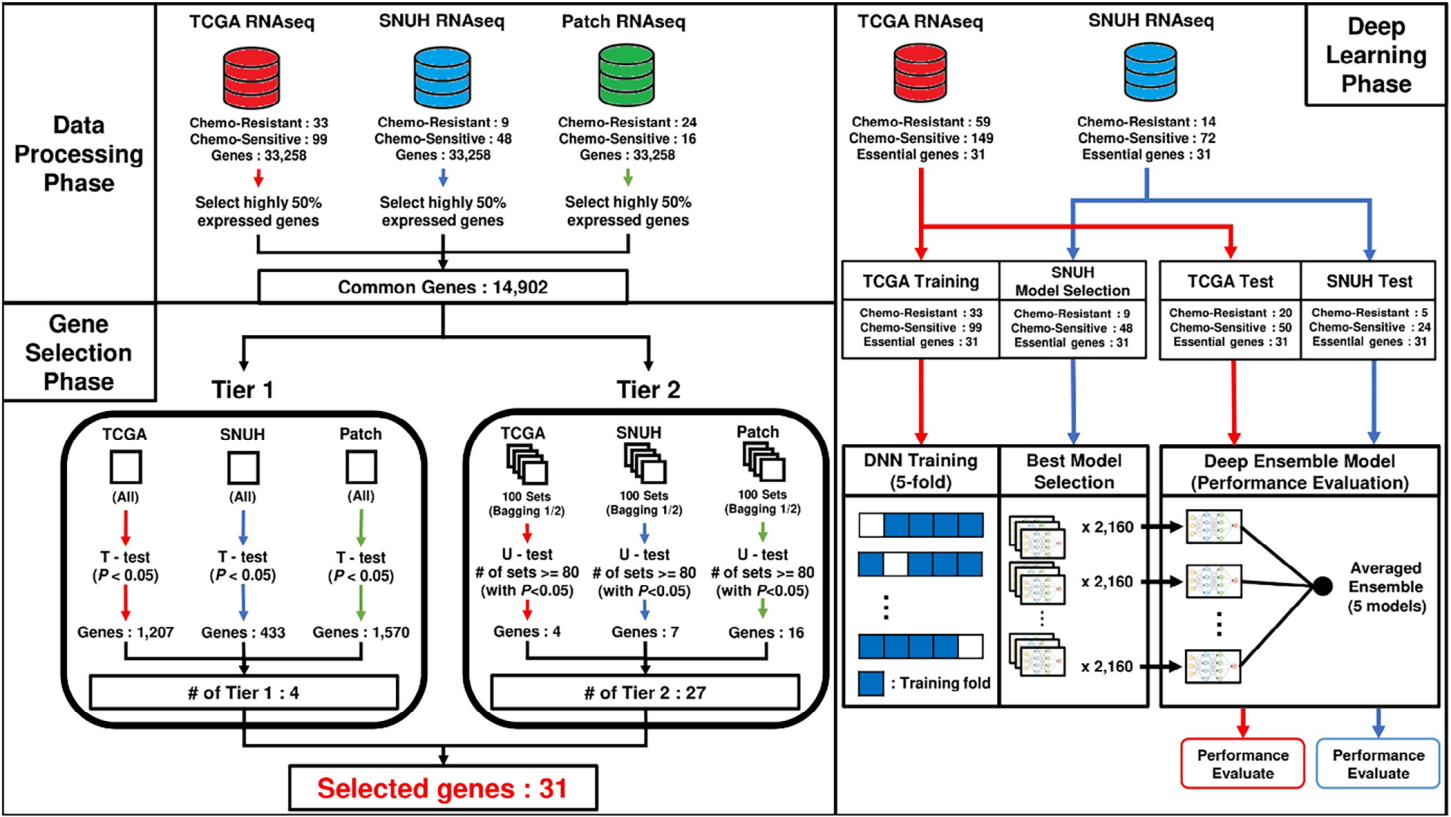
Overall workflow of the study analyses.

We aligned TCGA and SNUH fastq files to GRCh38 using HISAT2.0, yielding TPM gene expression data. Patch provided TPM data exclusively. Combining TCGA, SNUH and Patch TPM with ensemble IDs, we filtered out lowly expressed genes, resulting in 14 902 ensemble IDs. Each dataset was split 2:1 for training and testing, ensuring a balance between chemo‐resistant and chemo‐sensitive cases.(See Figure 2)

**FIGURE 2:**
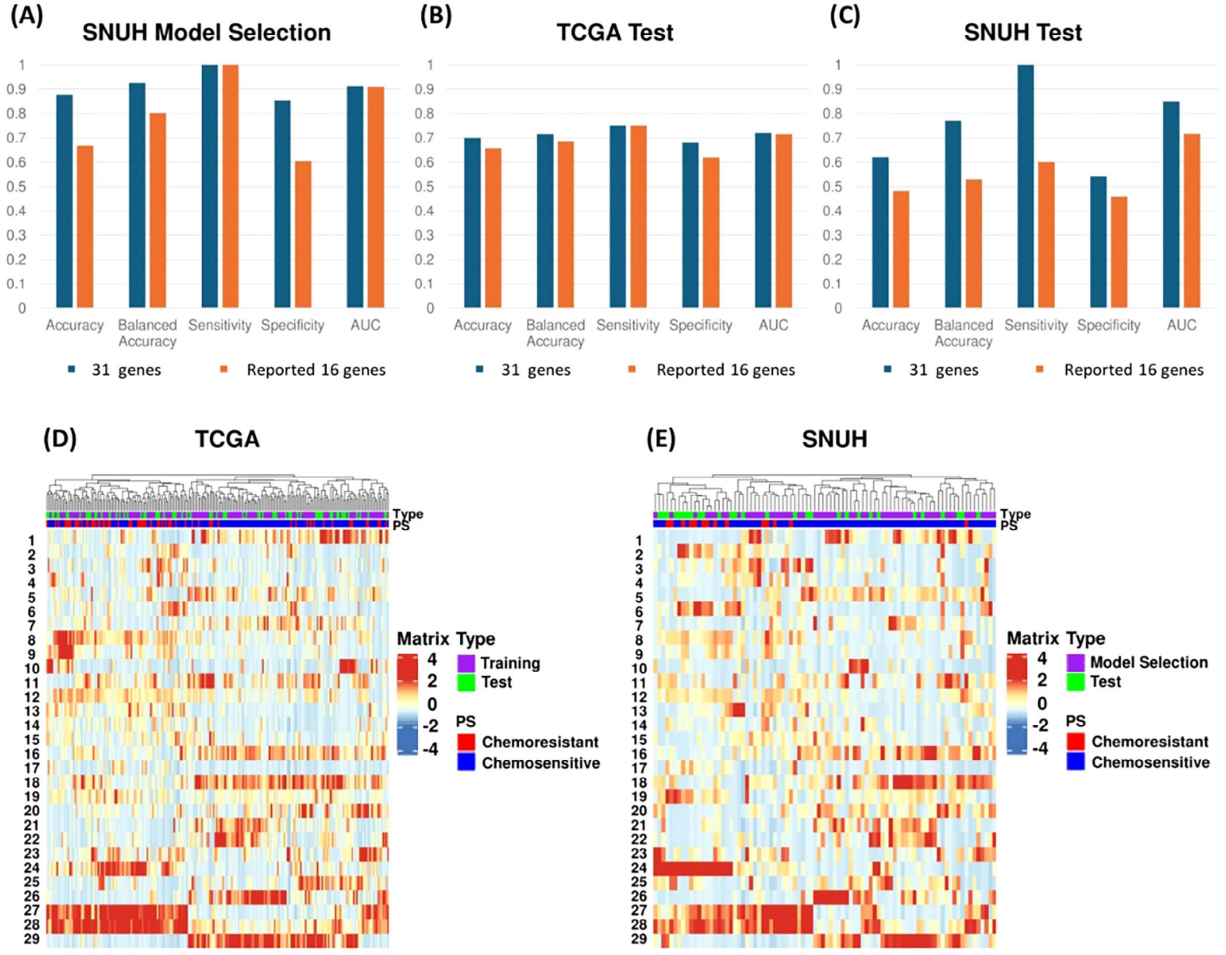
Predictive performance and visualization of deep ensemble model trained using 31 genes.

For gene selection, we used two strategies. The first aimed to capture the most concordant features across all datasets. A student's t‐tests were conducted for each gene, selecting those with a *p*‐value < .05 for each dataset. The intersection of these lists yielded four genes (tier1).

The second strategy involves identifying genes differentially expressed in each dataset. After 100 bagging trials with a balanced number of chemo‐resistant and chemo‐sensitive samples, genes were selected if significant in over 80 trials by Mann‐Whitney U‐tests (*p*‐value < .05), yielding 27 genes (4, 7 and 16 genes from TCGA, SNUH and Patch, respectively) (tier2). Combining these with the initial four genes (tier1) resulted in 31 genes for predicting chemo‐resistance (Tables S1).

TCGA training samples were split into five folds, ensuring class balance. Each fold underwent training with 2160 hyperparameter combinations using the Adam optimizer and binary cross‐entropy loss (Table S4). Models from each fold were applied to SNUH training data to select the best‐performing one (Table S5). The output values of these selected five models were averaged for predicting chemo‐resistance.

The deep ensemble model achieved AUCs of 0.721 and 0.85 for TCGA and SNUH, with sensitivities of 0.75 and 1.0, and specificities of 0.68 and 0.541 respectively. Another model using 16 previously reported genes yielded AUCs of 0.716 and 0.717 for TCGA and SNUH, with sensitivities of 0.75 and 0.6, and specificities of 0.62 and 0.458 respectively. Our selected 31 genes outperform the previous ones using the same method and data. Additionally, the 31 genes exhibit significantly higher AUC than models generated from randomly drawn the same number of genes. (Figure S1). These findings indicate both the increased number of genes and their potential biological relevance contribute to improved performance.

Visualization of the information that is held by the last layer of our deep ensemble model shows consistent chemo‐resistance classification performance across TCGA and SNUH datasets, despite ethnic composition differences between the datasets (Figure S2).

The 31 identified genes show 100% sensitivity in Koreans. Among them, the network of four (tier1) genes highlights pathways like “Cell Cycle: G1/S Checkpoint Regulation” and “DNA Methylation and Transcriptional Repression Signaling.” Key genes include TP53, E2F1, E2F4, HDAC1, HDAC2 and MYC1 (Figure S3A and Table S6). TP53 mutations induce chemotherapy resistance by targeting p53 complexes for therapy.^9^

E2F predicts chemoresistance, with histone deacetylases under study in ovarian cancer trials. MYC1 upregulated in chemo‐resistant ovarian cancer cells. Among the functions of 31 genes, the ‘Ribonucleotide Reductase Signaling Pathway’ stands out, including TP53, E2F, CDK4 and CREB1, suggesting functional coupling between tier1 and tier2 genes. (Figure S3B and Table S7). Targeting this pathway restores chemo‐sensitivity in chemo‐resistant ovarian cancer. CDK4 inhibition effectively restores chemo‐sensitivity in vivo, while inhibiting CREB1 phosphorylation sensitizes chemo‐resistant cells to platinum, crucial for preventing tumor recurrence.^10^

This study developed a deep ensemble model to predict chemoresistance in HGSOC patients. To compensate for the limited sample size of HGSOC patient data, we combine publicly available data with newly collected samples. With the strategy of combining common features in all data and features found in each data source, we identified 31 genes for predicting chemo‐resistant in this population. These genes achieved 100% sensitivity, 54.1% specificity and AUC 0.85 in the validation dataset and have documented roles in cases of ovarian cancer chemo‐resistant. The approach may be useful to build a prediction model with a limited sample size in conjunction with public resources. Especially, the identified genes and prediction models are worthy to be highlighted for further research to understand the biological significance and their application in other ovarian cancer research with a limited sample size.

## AUTHOR CONTRIBUTIONS

Se Ik Kim, Sangick Park and Eunyong Ahn contributed equally to this work. Se Ik Kim, Taejin Ahn and Yong Sang Song designed the study; Sangick Park and Jeunhui Kim analyzed the data and developed the prediction model; Se Ik Kim, Sangick Park, HyunA Jo, Juwon Lee, Untack Cho, Maria Lee, Cheol Lee and Danny N. Dhanasekaran collected pathological and clinical data; Cheol Lee reviewed and confirmed the pathological condition; Maria Lee, Cheol Lee, Danny N. Dhanasekaran and TP provided suggestions for the manuscript analysis results; Se Ik Kim and Sangick Park wrote the first draft of the manuscript; Eunyong Ahn reviewed the manuscript and wrote the final version of the manuscript; Taejin Ahn and Yong Sang Song supervised the research; all authors reviewed the manuscript, and approved the final report.

## CONFLICT OF INTEREST STATEMENT

The authors declare no conflict of interest.

## FUNDING INFORMATION

This work was supported by a grant from the Korea Health Technology R&D Project through the Korea Health Industry Development Institute (KHIDI), Republic of Korea (No. HI16C2037) and Korean National Research Foundation (NRF‐2019R1C1C1008185, 2022R1F1A1073939).

## ETHICS STATEMENT

This study was approved by the Institutional Review Board of SNUH (No. H‐1807‐037‐956). We conducted this study in accordance with the Declaration of Helsinki. All patients in the SNUH cohort provided written informed consent and donated their cancer tissues for scientific purposes.

## Supporting information

Supporting Information

## Data Availability

The RNA‐seq data from the TCGA are available via: https://portal.gdc.cancer.gov/projects/TCGA‐OV. The RNA‐seq gene expression data from Patch et al.’s study are accessible at the European Genome‐phenome Archive (EGA) data https://dcc.icgc.org/projects/. In addition, the RNA sequencing data from the SNUH cohort contains patients' sensitive information; therefore, its usage is possible after consulting with the corresponding author (Corresponding author(s). E‐mail(s): taejin.ahn@handong.edu; yssong@snu.ac.kr).
