# Supplementary material for: Tailored chemotherapy: Innovative deep‐learning model customizing chemotherapy for high‐grade serous ovarian carcinoma
Source: Clin Transl Med. 2024 Sep 7;14(9):e1774. doi: 10.1002/ctm2.1774 (PMC11380048; doi:10.1002/ctm2.1774)
Supplement: Supplementary file 1 — Supporting Information [file CTM2-14-e1774-s001.docx]

Previous submission number: CTM2-2024-03-0889

**Tailored Chemotherapy: Innovative Deep-Learning Model Customizing Chemotherapy for High-Grade Serous Ovarian Carcinoma**

Se Ik Kim^1†^, Sangick Park^2†^, Eunyong Ahn^3†^, Jeunhui Kim^2^, HyunA Jo^4,5^, Juwon Lee^4,5^, Untack Cho^4,6^, Maria Lee^1^, Cheol Lee^7^, Danny N. Dhanasekaran^8^, Taejin Ahn^3,9*^, Yong Sang Song^10*^

^1^Department of Obstetrics and Gynecology, Seoul National University College of Medicine, Seoul, 03080, Republic of Korea.

^2^Department of Advanced Green Energy and Environment, Handong Global University, Pohang, 37554, Republic of Korea.

^3^Foretell My Health, Pohang, 37554, Republic of Korea

^4^Cancer Research Institute, Seoul National University College of Medicine, Seoul, 03080, Republic of Korea.

^5^WCU Biomodulation, Department of Agricultural Biotechnology, Seoul National University, Seoul, 03080, Republic of Korea.

^6^Interdisciplinary Program in Cancer Biology, Seoul National University College of Medicine, Seoul, 03080, Republic of Korea.

^7^Department of Pathology, Seoul National University College of Medicine, Seoul, 03080, Republic of Korea

^8^Stephenson Cancer Center, University of Oklahoma Health Sciences Center, Oklahoma City, OK 73104, USA.

^9^Department of Life Science, Handong Global University, Pohang, 37554, Korea

^10^Department of Obstetrics and Gynaecology, Myongji Hospital, Hanyang University College of Medicine, Goyang, 10475, Republic of Korea

*Corresponding authors. E-mail(s): taejin.ahn@handong.edu; yssong@snu.ac.kr;

†Se Ik Kim, Sangick Park and Eunyong Ahn contributed equally to this work;

**ACKNOWLEDGEMENTS**

The biospecimens for this study were provided by the Seoul National University Hospital Cancer Tissue Bank, a member of Korea Biobank Network. All samples derived from the Seoul National University Hospital Cancer Tissue Bank were obtained with informed consent under institutional review board-approved protocols.

**Supplementary Figures**

**Fig. S1.** Comparison of predictive performance between 31 genes and randomly selected 31 genes in TCGA test and SNUH test.

**Fig. S2.** Clustering of the chemo-response group and visualization using the expression level of 31 genes in TCGA and SNUH datasets.

**Fig. S3.** Ingenuity Pathway Analysis (IPA) network plot of tire1 genes and 31 genes.

**Supplementary Tables**

**Table S1.** Patients’ clinicopathologic characteristics

**Table S2.** Log2 fold change and student’s T-test p-value of tier1 genes for TCGA, SNUH and Patch datasets.

**Table S3.** Sub-sample set counts with significant difference (P<0.05) of tier2 genes for TCGA and SNUH datasets.

**Table S4.** Hyper-parameters for deep learning model training.

**Table S5.** Hyper-parameters for best deep learning of each training fold.

**Table S6.** Ingenuity pathway analysis result of tier1 genes.

**Table S7.** Ingenuity pathway analysis result of 31 genes.


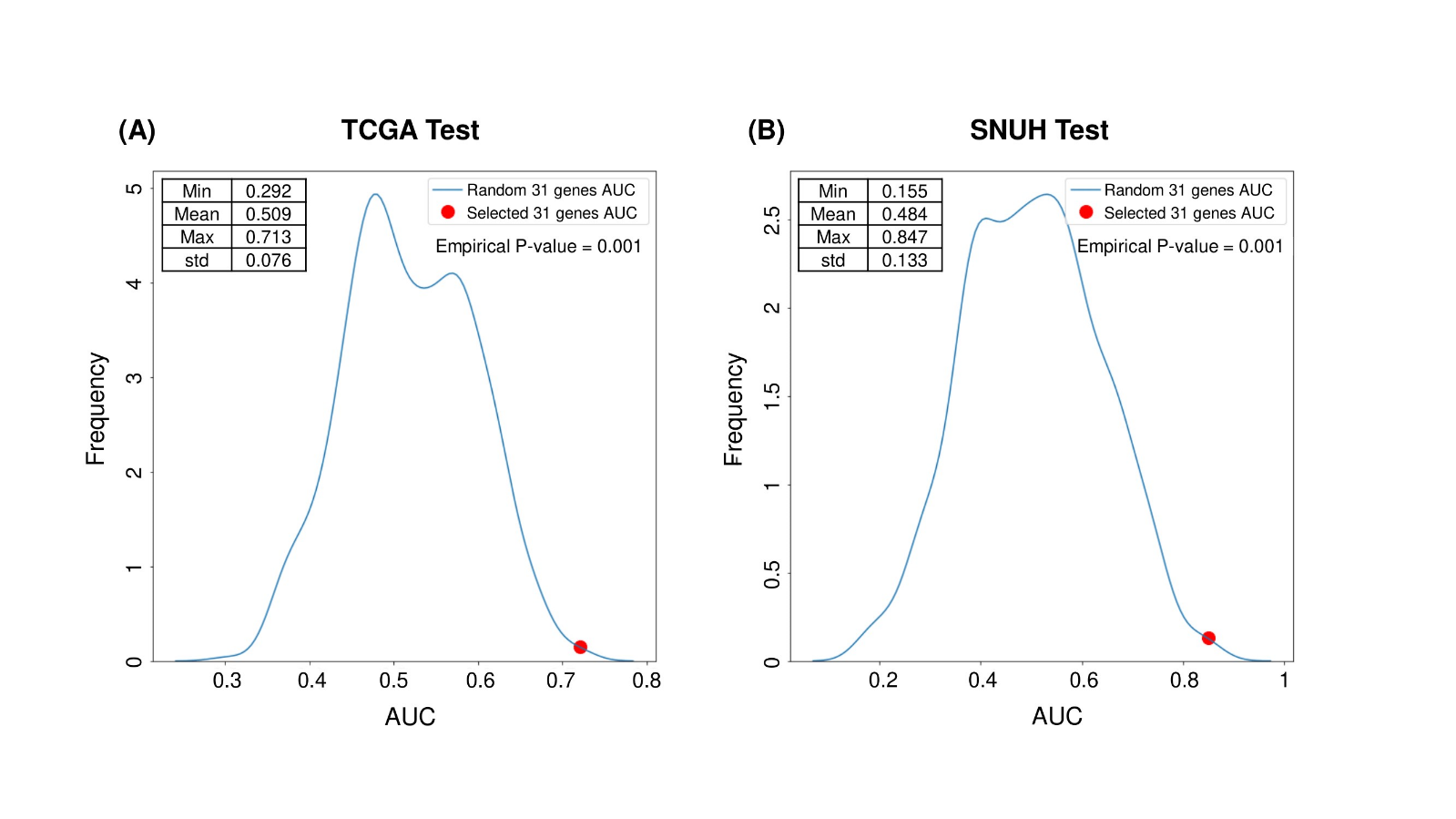


**Fig S1. Comparison of predictive performance between 31 genes and randomly selected 31 genes in TCGA test and SNUH test.**

This figure shows the performance of the deep learning model trained with each gene set. The distribution shown in the blue line and the red dot show the performance of the deep learning model trained with 1000 different random 31 genes and 31 genes, respectively. The x-axis represents the performance of the deep learning model, and the y-axis represents the frequency of the deep learning model performance. In table, the minimum, mean, maximum, and standard deviation values of each distribution are listed. **(A)** Comparison of performance of deep ensemble models trained with random 31 genes and 31 genes in TCGA test. The TCGA test AUCs of the deep ensemble model trained on 1000 sets of random 31 genes exhibited minimum, mean, maximum and standard deviation values of 0.292, 0.509, 0.713 and 0.076 respectively **(B)** Comparison of performance of deep ensemble models trained with random 31 genes and 31 genes in SNUH test. The SNUH test AUCs of the deep ensemble model trained on 1000 sets of random 31 genes exhibited minimum, mean, maximum and standard deviation values of 0.155, 0.484, 0.847 and 0.33 respectively.


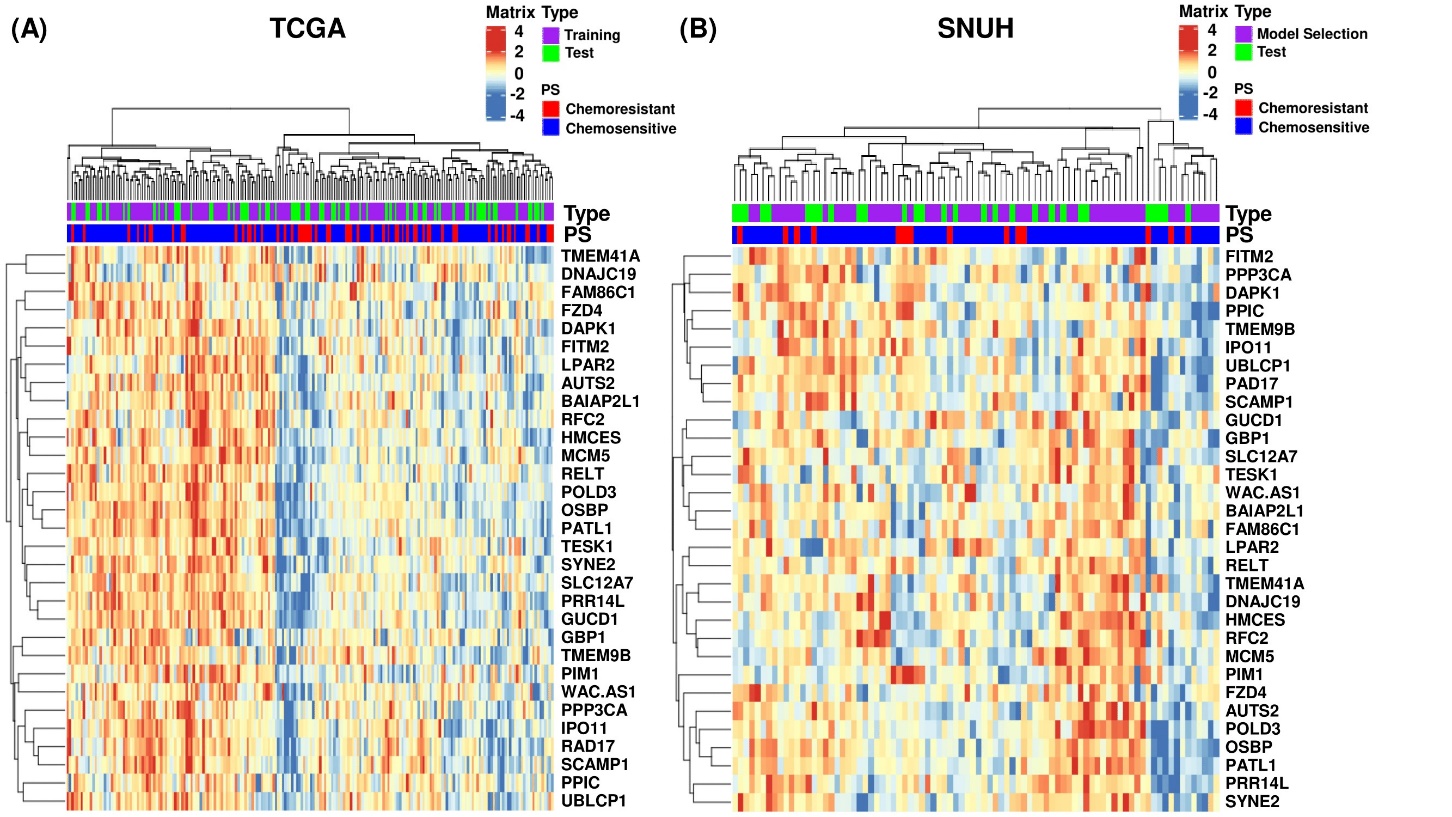


**Fig. S2. Clustering of the chemo-response group and visualization using the expression level of 31 genes in TCGA and SNUH datasets.**

The heatmap was accompanied by a dendrogram created using the complete distance calculation method for inter-sample expression of the 31 genes. The expression levels of these genes were row-scaled within the range of -4 to 4, where intense red signifies higher expression and intense blue represents lower expression. Chemoresistant samples are marked in red, while chemosensitive samples are indicated in blue on the PS column. (A) Represents a heatmap generated using the 31 genes from TCGA. Training samples are highlighted in purple, while test samples are indicated in green within the Type column. (B) Displays a heatmap created using the 31 genes from SNUH. Model selection samples are depicted in purple, and test samples are shown in green within the Type column.


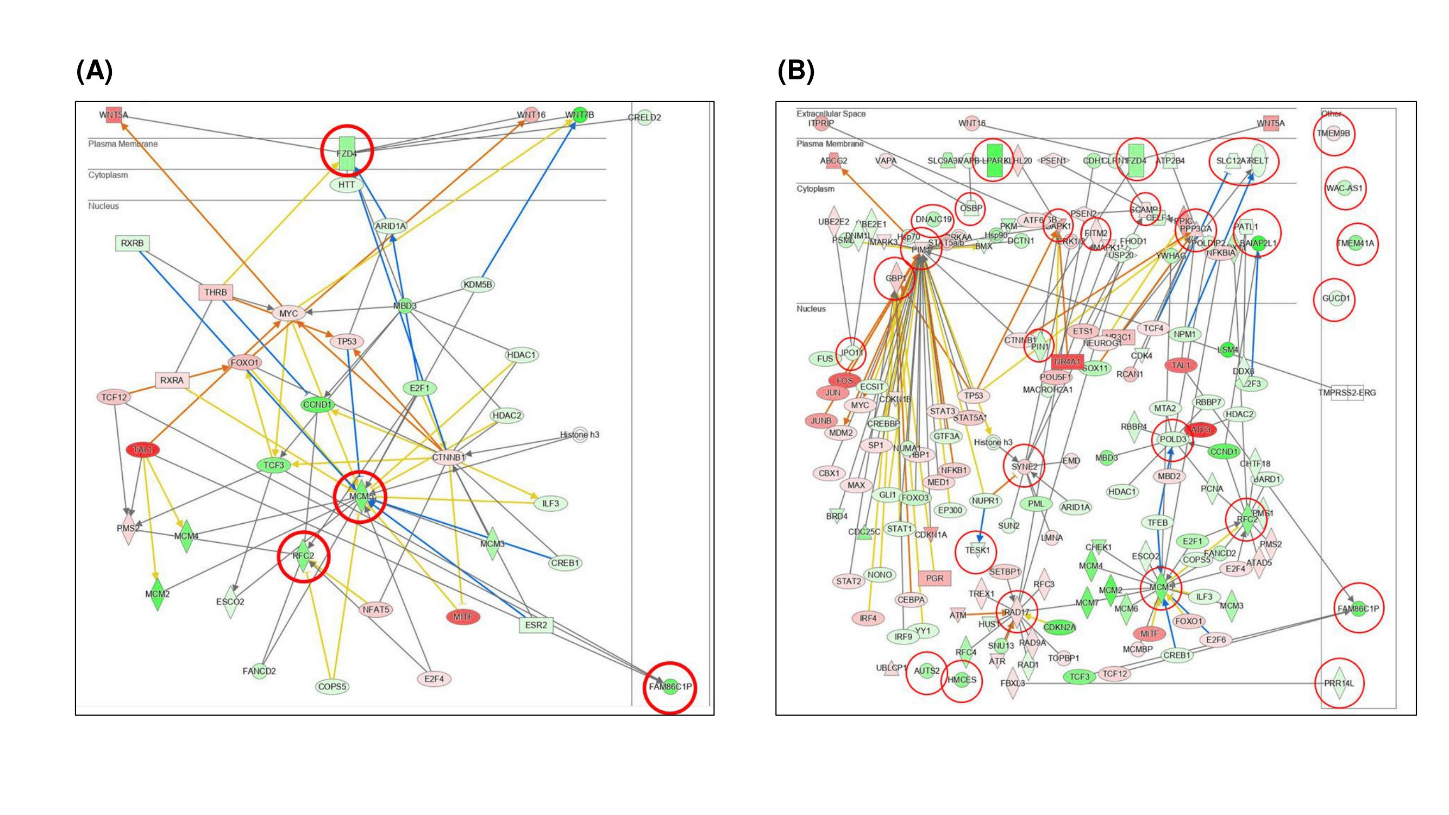


**Fig. S3. Ingenuity Pathway Analysis (IPA) network plot of tier1 genes and 31 genes.**

This figure depicts the results of the Ingenuity Pathway Analysis (IPA) presented as a network involving tier1 genes, 31 genes, their associated gene sets, and their placement based on the actual locations within the cellular environment. Each gene is represented by a shape, and the regulatory relationships between genes are indicated by arrows. The shapes with red color indicate up-regulated genes in the chemoresistant group, while shapes with green color indicate down-regulated genes, with darker colors indicating larger fold changes between the groups. Circular genes typically represent signaling molecules, factors, or proteins. Square genes denote transcription factors or elements regulating gene expression. Rhomboid-shaped genes indicate genes that activate or suppress functions. The tier1 genes and 31 genes are highlighted with red circles in each diagram. (A) IPA network for tier1 genes and related gene sets. (B) IPA network for 31 genes and related gene sets.

**Table S1. Patients’ clinicopathologic characteristics**

| **Characteristics** | **All**  **(n=86, %)** | **Chemosensitive**  **(n=72, %)** | **Chemoresistant**  **(n=14, %)** | ***P*** |
| --- | --- | --- | --- | --- |
| Age, years | 55.1 ± 11.1 | 54.9 ± 10.7 | 56.0 ± 13.7 | 0.788 |
| BMI, kg/m2 | 23.3 ± 3.5 | 23.2 ± 3.4 | 23.9 ± 3.9 | 0.699 |
| Parity |  |  |  | 0.844 |
| 0 | 11 (12.8) | 9 (12.5) | 3 (14.3) |  |
| 1–2 | 49 (57.0) | 42 (58.3) | 7 (50.0) |  |
| ≥3 | 26 (30.2) | 21 (29.2) | 5 (35.7) |  |
| Menopause | 53 (61.6) | 44 (61.1) | 9 (64.3) | 0.823 |
| Comorbidities |  |  |  |  |
| Hypertension | 19 (22.1) | 16 (22.2) | 3 (21.4) | >0.999 |
| Diabetes | 4 (4.7) | 3 (4.2) | 1 (7.1) | 0.516 |
| Dyslipidemia | 12 (14.0) | 9 (12.5) | 3 (21.4) | 0.404 |
| Serum CA-125, IU/mL | 841.0 (20.0–10000.0) | 841.0 (20.0–10000.0) | 798.5 (47.0–3628.0) | 0.657 |
| FIGO stage |  |  |  | 0.763 |
| IIIA-B | 11 (12.8) | 10 (13.9) | 1 (7.1) |  |
| IIIC | 54 (62.8) | 45 (62.5) | 9 (64.3) |  |
| IV | 21 (24.4) | 17 (23.6) | 4 (28.6) |  |
| Residual tumor size after CRS |  |  |  | 0.056 |
| Complete resection | 60 (69.8) | 54 (75.0) | 6 (42.9) |  |
| Residual <1 cm | 19 (22.1) | 13 (18.1) | 6 (42.9) |  |
| Residual ≥1 cm | 7 (8.1) | 5 (6.9) | 2 (14.3) |  |
| First-line chemotherapy regimen |  |  |  | >0.999 |
| Paclitaxel-Carboplatin | 79 (91.9) | 66 (91.7) | 13 (92.9) |  |
| Paclitaxel-Carboplatin-BEV | 7 (8.1) | 6 (8.3) | 1 (7.1) |  |
| Total cycle of first-line chemotherapy |  |  |  | 0.450 |
| 6 | 72 (83.7) | 59 (81.9) | 13 (92.9) |  |
| 9 | 14 (16.3) | 13 (18.1) | 1 (7.1) |  |
| Platinum-free interval, months | 19.6 (0.4–92.5) | 24.5 (6.6–92.5) | 3.4 (0.4–5.8) | <0.001 |
| Germline *BRCA1/2* status |  |  |  |  |
| Not performed | 28 (32.6) | 22 (30.6) | 6 (42.9) | 0.370 |
| Performed | 58 (67.4) | 50 (69.4) | 8 (57.1) |  |
| No mutation | 41 (47.7) | 34 (47.2) | 7 (50.0) | 0.479 |
| *BRCA1* mutation | 12 (14.0) | 11 (15.3) | 1 (7.1) |  |
| *BRCA2* mutation | 5 (5.8) | 5 (6.9) | 0 |  |
| Both | 0 | 0 | 0 |  |
| Data are presented as mean ± standard deviation or median (range) for continuous variables and as count (%) for categorical variables.  Abbreviations: BEV, bevacizumab; BMI, body mass index; CA-125, cancer antigen 125; CRS, cytoreductive surgery; FIGO, International Federation of Gynecology and Obstetrics. | | | | |

Table S2. Log2 fold change and student’s T-test p-value of tier1 genes for TCGA, SNUH and Patch datasets.

| Gene | Log2 FC  (Res/Sen) | *P*-value  in TCGA | Log2 FC  (Res/Sen) | *P*-value  in SNUH | Log2 FC  (Res/Sen) | *P*-value  in Patch |
| --- | --- | --- | --- | --- | --- | --- |
| RFC2 | -0.563 | 2.719.E-03 | -0.738 | 2.570.E-02 | -0.686 | 9.841.E-03 |
| MCM5 | -0.435 | 1.988.E-02 | -0.843 | 4.342.E-02 | -0.652 | 1.644.E-02 |
| FAM86C1 | -0.489 | 1.521.E-02 | -0.687 | 4.263.E-02 | -0.582 | 3.539.E-02 |
| FZD4 | -0.507 | 6.774.E-03 | -0.602 | 1.196.E-02 | -0.688 | 1.431.E-02 |

Abbreviations: fold change; FC, chemoresistant; Res, chemosensitive; Sen

Table S3. Sub-sample set counts with significant difference (P<0.05) of tier2 genes for TCGA and SNUH datasets.

| Dataset | Gene | # of sub-sample sets (with *P*<0.05) |
| --- | --- | --- |
| TCGA | PRR14L | 86 |
|  | OSBP | 81 |
|  | GUCD1 | 96 |
|  | PATL1 | 98 |
| SNUH | TMEM41A | 80 |
|  | DNAJC19 | 80 |
|  | HMCES | 92 |
|  | AUTS2 | 93 |
|  | BAIAP2L1 | 90 |
|  | LPAR2 | 96 |
|  | PPIC | 87 |
| Patch | SLC12A7 | 97 |
|  | GBP1 | 97 |
|  | POLD3 | 88 |
|  | PIM1 | 87 |
|  | SYNE2 | 84 |
|  | WAC-AS1 | 89 |
|  | PPP3CA | 95 |
|  | DAPK1 | 85 |
|  | IPO11 | 81 |
|  | RELT | 100 |
|  | FITM2 | 89 |
|  | UBLCP1 | 80 |
|  | TMEM9B | 90 |
|  | TESK1 | 83 |
|  | RAD17 | 81 |
|  | SCAMP1 | 87 |

**Table S4.** **Hyper-parameters for deep learning model training.**

| List of hyper-parameters | |
| --- | --- |
| Loss function | Binary Cross Entropy |
| Optimizer | Adam |
| Hidden layers | [15, 7, 5, 3, 3]  [15, 7, 5, 3]  [15, 7, 5]  [15, 7] |
| Learning rate | [0.01, 0.001, 0.0001] |
| Batch size | [10, 12, 15, 17, 20] |
| Drop-out rate | [0, 0.1, 0.2, 0.3, 0.4, 0.5, 0.6, 0.7, 0.8] |
| Early stopping patience | [5, 10, 15, 20] |

**Table S5. Hyper-parameters for best deep learning of each training fold.**

| **Training**  **fold** | **Loss**  **function** | **Optimizer** | **Learning**  **rate** | **Early stopping**  **patience** | **Hidden**  **Layers** | **Batch size** | **Dropout**  **rate** |
| --- | --- | --- | --- | --- | --- | --- | --- |
| 1 | Binary cross entropy | Adam | 0.0001 | 5 | [15, 7] | 20 | 0.4 |
| 2 |  |  | 0.0001 | 20 | [15, 7, 5] | 20 | 0.1 |
| 3 |  |  | 0.0001 | 20 | [15, 7] | 10 | 0.7 |
| 4 |  |  | 0.0001 | 20 | [15, 7] | 12 | 0.2 |
| 5 |  |  | 0.001 | 5 | [15, 7, 5, 3] | 15 | 0.8 |

**Table S6. Ingenuity pathway analysis result of tier1 genes.**

| Ingenuity Canonical Pathways | p-value | z-score | Activation |
| --- | --- | --- | --- |
| Ovarian Cancer Signaling | 6.310E-19 |  | - |
| Chronic Myeloid Leukemia Signaling | 1.995E-17 | -5.350E-01 | Negative |
| Glioblastoma Multiforme Signaling | 1.000E-16 | 0.000E+00 | - |
| Molecular Mechanisms of Cancer | 6.310E-16 |  | - |
| Small Cell Lung Cancer Signaling | 7.943E-14 |  | - |
| DNA Methylation and Transcriptional Repression Signaling | 3.162E-13 | -8.160E-01 | Negative |
| Cell Cycle: G1/S Checkpoint Regulation | 3.162E-13 | 1.414E+00 | Positive |
| Prostate Cancer Signaling | 3.162E-13 |  | - |
| WNT/β-catenin Signaling | 3.981E-13 | 6.320E-01 | Positive |
| Hereditary Breast Cancer Signaling | 2.512E-12 |  | - |
| Factors Promoting Cardiogenesis in Vertebrates | 5.012E-12 | -3.330E-01 | Negative |
| Non-Small Cell Lung Cancer Signaling | 5.012E-12 |  | - |
| HOTAIR Regulatory Pathway | 7.943E-12 | -3.330E-01 | Negative |
| Colorectal Cancer Metastasis Signaling | 3.162E-11 | -6.320E-01 | Negative |
| Basal Cell Carcinoma Signaling | 3.981E-11 | 0.000E+00 | - |
| Role of Macrophages, Fibroblasts and Endothelial Cells in Rheumatoid Arthritis | 1.820E-10 |  | - |
| Ribonucleotide Reductase Signaling Pathway | 6.026E-10 | -7.070E-01 | Negative |
| Estrogen-mediated S-phase Entry | 9.550E-10 | -4.470E-01 | Negative |
| Bladder Cancer Signaling | 1.288E-09 |  | - |
| Pulmonary Healing Signaling Pathway | 2.042E-09 | 0.000E+00 | - |
| Human Embryonic Stem Cell Pluripotency | 2.042E-09 | 1.414E+00 | Positive |
| Hepatic Fibrosis Signaling Pathway | 2.291E-09 | 3.330E-01 | Positive |
| Role of WNT/GSK-3β Signaling in the Pathogenesis of Influenza | 3.981E-09 | 4.470E-01 | Positive |
| Adipogenesis pathway | 4.677E-09 | -3.780E-01 | Negative |
| Pulmonary Fibrosis Idiopathic Signaling Pathway | 4.677E-09 | -3.330E-01 | Negative |
| Thyroid Cancer Signaling | 5.012E-09 | 0.000E+00 | - |
| Role of Osteoblasts, Osteoclasts and Chondrocytes in Rheumatoid Arthritis | 5.129E-09 |  | - |
| Role of BRCA1 in DNA Damage Response | 6.310E-09 | 0.000E+00 | - |
| Aryl Hydrocarbon Receptor Signaling | 7.586E-09 | -1.134E+00 | Negative |
| Cyclins and Cell Cycle Regulation | 8.511E-09 | -1.000E+00 | Negative |
| Regulation Of The Epithelial Mesenchymal Transition In Development Pathway | 9.120E-09 | 4.470E-01 | Positive |
| CDX Gastrointestinal Cancer Signaling Pathway | 5.370E-08 | 3.780E-01 | Positive |
| Role of NANOG in Mammalian Embryonic Stem Cell Pluripotency | 8.128E-08 |  | - |
| Glioma Signaling | 8.128E-08 |  | - |
| Myelination Signaling Pathway | 8.913E-08 | -1.414E+00 | Negative |
| Pancreatic Adenocarcinoma Signaling | 8.913E-08 |  | - |
| Cardiac Hypertrophy Signaling (Enhanced) | 3.311E-07 | 1.667E+00 | Positive |
| S100 Family Signaling Pathway | 6.026E-07 | -1.265E+00 | Negative |
| p53 Signaling | 8.913E-07 |  | - |
| Mouse Embryonic Stem Cell Pluripotency | 1.148E-06 | -4.470E-01 | Negative |
| Regulation of the Epithelial-Mesenchymal Transition Pathway | 1.202E-06 |  | - |
| Coronavirus Pathogenesis Pathway | 1.259E-06 | 1.633E+00 | Positive |
| Telomerase Signaling | 1.259E-06 |  | - |
| Melanoma Signaling | 1.995E-06 |  | - |
| Cell Cycle Control of Chromosomal Replication | 3.162E-06 | -2.000E+00 | Negative |
| Role Of Osteoblasts In Rheumatoid Arthritis Signaling Pathway | 3.162E-06 | 0.000E+00 | - |
| Role of CHK Proteins in Cell Cycle Checkpoint Control | 3.631E-06 |  | - |
| PCP (Planar Cell Polarity) Pathway | 4.169E-06 | 0.000E+00 | - |
| Endometrial Cancer Signaling | 4.169E-06 |  | - |
| MSP-RON Signaling In Cancer Cells Pathway | 5.012E-06 | -4.470E-01 | Negative |
| WNT/Ca+ pathway | 5.754E-06 | 0.000E+00 | - |
| Endocannabinoid Cancer Inhibition Pathway | 6.310E-06 | 4.470E-01 | Positive |
| ID1 Signaling Pathway | 2.951E-05 | -4.470E-01 | Negative |
| Sumoylation Pathway | 3.311E-05 |  | - |
| Autophagy | 3.981E-05 | -4.470E-01 | Negative |
| Cell Cycle Regulation by BTG Family Proteins | 4.467E-05 |  | - |
| Osteoarthritis Pathway | 5.888E-05 | -4.470E-01 | Negative |
| BER (Base Excision Repair) Pathway | 6.918E-05 |  | - |
| Role of OCT4 in Mammalian Embryonic Stem Cell Pluripotency | 7.413E-05 |  | - |
| Estrogen Receptor Signaling | 7.762E-05 | -1.633E+00 | Negative |
| White Adipose Tissue Browning Pathway | 1.072E-04 | 0.000E+00 | - |
| Huntington's Disease Signaling | 1.380E-04 |  | - |
| Polyamine Regulation in Colon Cancer | 1.660E-04 |  | - |
| GADD45 Signaling | 1.660E-04 |  | - |
| Role Of Osteoclasts In Rheumatoid Arthritis Signaling Pathway | 1.738E-04 | -4.470E-01 | Negative |
| Senescence Pathway | 1.820E-04 | 1.342E+00 | Positive |
| ERB2-ERBB3 Signaling | 2.138E-04 |  | - |
| Axonal Guidance Signaling | 2.344E-04 |  | - |
| IL-7 Signaling Pathway | 3.162E-04 |  | - |
| Hypoxia Signaling in the Cardiovascular System | 3.236E-04 |  | - |
| MicroRNA Biogenesis Signaling Pathway | 3.311E-04 | 1.000E+00 | Positive |
| VDR/RXR Activation | 3.631E-04 |  | - |
| Macrophage Alternative Activation Signaling Pathway | 3.981E-04 | 0.000E+00 | - |
| Glucocorticoid Receptor Signaling | 3.981E-04 |  | - |
| ILK Signaling | 4.266E-04 | 0.000E+00 | Positive |
| BEX2 Signaling Pathway | 4.266E-04 |  | - |
| PI3K/AKT Signaling | 4.467E-04 |  | - |
| TR/RXR Activation | 4.677E-04 |  | - |
| Mismatch Repair in Eukaryotes | 4.786E-04 |  | - |
| Calcium Signaling | 5.623E-04 |  | - |
| Acute Myeloid Leukemia Signaling | 5.754E-04 |  | - |
| Breast Cancer Regulation by Stathmin1 | 5.888E-04 | -1.633E+00 | Negative |
| HER-2 Signaling in Breast Cancer | 6.918E-04 | -1.000E+00 | Negative |
| ATM Signaling | 7.586E-04 |  | - |
| AMPK Signaling | 9.333E-04 |  | - |
| Chaperone Mediated Autophagy Signaling Pathway | 1.072E-03 | -4.470E-01 | Negative |
| Systemic Lupus Erythematosus In B Cell Signaling Pathway | 1.259E-03 | 4.470E-01 | Positive |
| Role of p14/p19ARF in Tumor Suppression | 1.259E-03 |  | - |
| p38 MAPK Signaling | 1.318E-03 |  | - |
| G-Protein Coupled Receptor Signaling | 1.380E-03 | -8.160E-01 | Negative |
| Opioid Signaling Pathway | 1.514E-03 |  | - |
| Endocannabinoid Developing Neuron Pathway | 1.585E-03 |  | - |
| CLEAR Signaling Pathway | 1.622E-03 | 1.000E+00 | Positive |
| Sirtuin Signaling Pathway | 1.698E-03 | 1.000E+00 | Positive |
| DNA damage-induced 14-3-3σ Signaling | 3.162E-03 |  | - |
| PFKFB4 Signaling Pathway | 3.388E-03 |  | - |
| FAK Signaling | 3.467E-03 | -8.160E-01 | Negative |
| Erythropoietin Signaling Pathway | 3.467E-03 |  | - |
| MYC Mediated Apoptosis Signaling | 3.715E-03 |  | - |
| FAT10 Cancer Signaling Pathway | 3.715E-03 |  | - |
| Tumor Microenvironment Pathway | 4.074E-03 |  | - |
| B Cell Receptor Signaling | 4.266E-03 |  | - |
| CSDE1 Signaling Pathway | 4.467E-03 |  | - |
| Retinoic acid Mediated Apoptosis Signaling | 4.786E-03 |  | - |
| Cancer Drug Resistance By Drug Efflux | 5.012E-03 |  | - |
| RAR Activation | 5.370E-03 |  | - |
| Protein Kinase A Signaling | 5.495E-03 |  | - |
| PXR/RXR Activation | 5.754E-03 |  | - |
| ERK5 Signaling | 7.943E-03 |  | - |
| Role of JAK family kinases in IL-6-type Cytokine Signaling | 8.511E-03 |  | - |
| Estrogen-Dependent Breast Cancer Signaling | 9.333E-03 |  | - |
| BAG2 Signaling Pathway | 9.550E-03 |  | - |
| PEDF Signaling | 1.023E-02 |  | - |
| NUR77 Signaling in T Lymphocytes | 1.349E-02 |  | - |
| Melanocyte Development and Pigmentation Signaling | 1.349E-02 |  | - |
| NER (Nucleotide Excision Repair, Enhanced Pathway) | 1.479E-02 |  | - |
| Neuroinflammation Signaling Pathway | 1.820E-02 |  | - |
| IL-13 Signaling Pathway | 1.820E-02 |  | - |
| NGF Signaling | 1.950E-02 |  | - |
| LXR/RXR Activation | 2.089E-02 |  | - |
| FXR/RXR Activation | 2.138E-02 |  | - |
| Mitochondrial Dysfunction | 2.188E-02 |  | - |
| Ferroptosis Signaling Pathway | 2.239E-02 |  | - |
| Role of PKR in Interferon Induction and Antiviral Response | 2.399E-02 |  | - |
| P2Y Purigenic Receptor Signaling Pathway | 2.399E-02 |  | - |
| Phospholipase C Signaling | 2.884E-02 | -1.000E+00 | Negative |
| IL-10 Signaling | 3.090E-02 |  | - |
| PTEN Signaling | 3.090E-02 |  | - |
| T Cell Receptor Signaling | 3.090E-02 |  | - |
| Epithelial Adherens Junction Signaling | 3.236E-02 |  | - |
| Regulation Of The Epithelial Mesenchymal Transition By Growth Factors Pathway | 4.677E-02 |  | - |
| Adrenomedullin signaling pathway | 4.786E-02 |  | - |

**Table S7. Ingenuity pathway analysis result of 31 genes.**

| Ingenuity Canonical Pathways | p-value | z-score | Activation |
| --- | --- | --- | --- |
| Chronic Myeloid Leukemia Signaling | 3.16E-28 | 3.54E-01 | Positive |
| Molecular Mechanisms of Cancer | 3.16E-28 | - | - |
| Ribonucleotide Reductase Signaling Pathway | 5.01E-26 | 3.92E-01 | Positive |
| Role of CHK Proteins in Cell Cycle Checkpoint Control | 2.51E-24 | 8.32E-01 | Positive |
| Cell Cycle: G1/S Checkpoint Regulation | 5.01E-23 | 1.41E+00 | Positive |
| Sumoylation Pathway | 1.59E-22 | 1.00E+00 | Positive |
| Hereditary Breast Cancer Signaling | 5.01E-21 | - | - |
| Aryl Hydrocarbon Receptor Signaling | 2.00E-20 | 2.18E-01 | Positive |
| DNA Methylation and Transcriptional Repression Signaling | 3.98E-20 | -1.29E+00 | Negative |
| Prostate Cancer Signaling | 3.98E-20 | - | - |
| Role of BRCA1 in DNA Damage Response | 7.94E-20 | 3.02E-01 | Positive |
| Senescence Pathway | 1.00E-19 | 1.57E+00 | Positive |
| p53 Signaling | 1.00E-19 | -5.77E-01 | Negative |
| HOTAIR Regulatory Pathway | 1.00E-19 | -8.94E-01 | Negative |
| ID1 Signaling Pathway | 1.26E-17 | -6.55E-01 | Negative |
| DNA damage-induced 14-3-3σ Signaling | 3.16E-17 | 8.32E-01 | Positive |
| Coronavirus Pathogenesis Pathway | 1.00E-16 | 2.24E+00 | Positive |
| ATM Signaling | 1.00E-16 | 2.14E+00 | Positive |
| Glioblastoma Multiforme Signaling | 1.26E-16 | 5.35E-01 | Positive |
| WNT/β-catenin Signaling | 1.59E-16 | 7.75E-01 | Positive |
| Pancreatic Adenocarcinoma Signaling | 2.00E-16 | 6.32E-01 | Positive |
| Ovarian Cancer Signaling | 5.01E-16 | - | - |
| Bladder Cancer Signaling | 1.00E-15 | - | - |
| Cyclins and Cell Cycle Regulation | 5.01E-15 | -1.73E+00 | Negative |
| Role of PKR in Interferon Induction and Antiviral Response | 1.00E-14 | 2.00E+00 | Positive |
| Pulmonary Fibrosis Idiopathic Signaling Pathway | 1.59E-14 | 4.26E-01 | Positive |
| MSP-RON Signaling In Cancer Cells Pathway | 2.51E-14 | 1.00E+00 | Positive |
| Small Cell Lung Cancer Signaling | 3.16E-14 | -1.00E+00 | Negative |
| Autophagy | 1.26E-13 | 1.41E+00 | Positive |
| HER-2 Signaling in Breast Cancer | 3.16E-13 | 1.41E+00 | Positive |
| Estrogen-mediated S-phase Entry | 3.16E-13 | -1.00E+00 | Negative |
| Pulmonary Healing Signaling Pathway | 5.01E-13 | 1.21E+00 | Positive |
| Hypoxia Signaling in the Cardiovascular System | 7.94E-13 | -1.00E+00 | Negative |
| GADD45 Signaling | 1.26E-12 | 9.05E-01 | Positive |
| IL-10 Signaling | 1.59E-12 | -1.29E+00 | Negative |
| Cell Cycle: G2/M DNA Damage Checkpoint Regulation | 6.31E-12 | 1.00E+00 | Positive |
| Melanoma Signaling | 6.31E-12 | -1.13E+00 | Negative |
| Endocannabinoid Cancer Inhibition Pathway | 1.26E-11 | 1.07E+00 | Positive |
| Non-Small Cell Lung Cancer Signaling | 1.26E-11 | -4.47E-01 | Negative |
| Myelination Signaling Pathway | 1.26E-11 | -6.88E-01 | Negative |
| Glucocorticoid Receptor Signaling | 1.26E-11 | - | - |
| Hepatic Fibrosis Signaling Pathway | 2.00E-11 | 1.34E+00 | Positive |
| Glioma Signaling | 2.00E-11 | - | - |
| Thyroid Cancer Signaling | 3.16E-11 | 1.51E+00 | Positive |
| Colorectal Cancer Metastasis Signaling | 5.01E-11 | 1.21E+00 | Positive |
| BER (Base Excision Repair) Pathway | 6.31E-11 | -3.33E-01 | Negative |
| Regulation Of The Epithelial Mesenchymal Transition In Development Pathway | 7.94E-11 | 1.67E+00 | Positive |
| Estrogen Receptor Signaling | 1.00E-10 | 6.88E-01 | Positive |
| Role of Macrophages, Fibroblasts and Endothelial Cells in Rheumatoid Arthritis | 1.10E-10 | - | - |
| Acute Myeloid Leukemia Signaling | 1.62E-10 | 1.27E+00 | Positive |
| Prolactin Signaling | 2.95E-10 | 2.12E+00 | Positive |
| BAG2 Signaling Pathway | 1.02E-09 | -3.33E-01 | Negative |
| ERB2-ERBB3 Signaling | 2.19E-09 | 1.13E+00 | Positive |
| Macrophage Alternative Activation Signaling Pathway | 5.89E-09 | 8.32E-01 | Positive |
| Regulation of the Epithelial-Mesenchymal Transition Pathway | 5.89E-09 | - | - |
| IL-7 Signaling Pathway | 7.24E-09 | -3.78E-01 | Negative |
| PI3K/AKT Signaling | 8.13E-09 | 9.05E-01 | Positive |
| Mouse Embryonic Stem Cell Pluripotency | 1.10E-08 | 6.32E-01 | Positive |
| Role of JAK family kinases in IL-6-type Cytokine Signaling | 1.18E-08 | 1.00E+00 | Positive |
| PPAR Signaling | 1.20E-08 | -1.90E+00 | Negative |
| Telomerase Signaling | 1.32E-08 | 4.47E-01 | Positive |
| Cell Cycle Control of Chromosomal Replication | 1.55E-08 | -2.83E+00 | Negative |
| Estrogen-Dependent Breast Cancer Signaling | 1.66E-08 | 1.00E+00 | Positive |
| BEX2 Signaling Pathway | 1.82E-08 | 3.33E-01 | Positive |
| JAK/STAT Signaling | 2.04E-08 | 1.67E+00 | Positive |
| Role of Osteoblasts, Osteoclasts and Chondrocytes in Rheumatoid Arthritis | 3.16E-08 | - | - |
| Activation of IRF by Cytosolic Pattern Recognition Receptors | 3.98E-08 | -1.41E+00 | Negative |
| CDX Gastrointestinal Cancer Signaling Pathway | 6.31E-08 | 1.73E+00 | Positive |
| iNOS Signaling | 6.92E-08 | 1.13E+00 | Positive |
| Chaperone Mediated Autophagy Signaling Pathway | 8.91E-08 | -2.43E-01 | Negative |
| Basal Cell Carcinoma Signaling | 9.33E-08 | 8.16E-01 | Positive |
| HIF1α Signaling | 1.05E-07 | 0.00E+00 | - |
| Role Of Osteoclasts In Rheumatoid Arthritis Signaling Pathway | 1.12E-07 | 1.60E+00 | Positive |
| Erythropoietin Signaling Pathway | 1.23E-07 | 1.51E+00 | Positive |
| MYC Mediated Apoptosis Signaling | 1.45E-07 | -3.78E-01 | Negative |
| Adipogenesis pathway | 1.70E-07 | 6.32E-01 | Positive |
| VDR/RXR Activation | 2.00E-07 | - | - |
| IL-3 Signaling | 2.40E-07 | 1.41E+00 | Positive |
| Mismatch Repair in Eukaryotes | 2.51E-07 | -4.47E-01 | Negative |
| Role Of Osteoblasts In Rheumatoid Arthritis Signaling Pathway | 3.47E-07 | 0.00E+00 | - |
| Factors Promoting Cardiogenesis in Vertebrates | 3.72E-07 | 0.00E+00 | - |
| Opioid Signaling Pathway | 3.80E-07 | 6.32E-01 | Positive |
| Cancer Drug Resistance By Drug Efflux | 4.17E-07 | 1.13E+00 | Positive |
| Osteoarthritis Pathway | 4.17E-07 | 6.32E-01 | Positive |
| Huntington's Disease Signaling | 4.27E-07 | - | - |
| Polyamine Regulation in Colon Cancer | 4.68E-07 | 1.89E+00 | Positive |
| Endometrial Cancer Signaling | 5.25E-07 | 1.63E+00 | Positive |
| Cell Cycle Regulation by BTG Family Proteins | 5.62E-07 | 8.16E-01 | Positive |
| ILK Signaling | 5.75E-07 | 9.05E-01 | Positive |
| Sirtuin Signaling Pathway | 5.75E-07 | -8.32E-01 | Negative |
| Human Embryonic Stem Cell Pluripotency | 6.03E-07 | 2.11E+00 | Positive |
| Thrombopoietin Signaling | 7.41E-07 | 1.89E+00 | Positive |
| Ferroptosis Signaling Pathway | 9.12E-07 | 1.67E+00 | Positive |
| MIF Regulation of Innate Immunity | 1.38E-06 | 1.63E+00 | Positive |
| GM-CSF Signaling | 1.51E-06 | -4.47E-01 | Negative |
| Role Of Chondrocytes In Rheumatoid Arthritis Signaling Pathway | 2.04E-06 | 2.33E+00 | Positive |
| Macrophage Classical Activation Signaling Pathway | 2.09E-06 | -1.13E+00 | Negative |
| Acute Phase Response Signaling | 2.51E-06 | 1.67E+00 | Positive |
| S100 Family Signaling Pathway | 2.75E-06 | 1.34E+00 | Positive |
| FAT10 Cancer Signaling Pathway | 2.95E-06 | 1.63E+00 | Positive |
| CLEAR Signaling Pathway | 3.09E-06 | 0.00E+00 | - |
| Role of p14/p19ARF in Tumor Suppression | 3.24E-06 | 0.00E+00 | - |
| FLT3 Signaling in Hematopoietic Progenitor Cells | 4.07E-06 | 3.78E-01 | Positive |
| RAR Activation | 4.47E-06 | - | - |
| MSP-RON Signaling In Macrophages Pathway | 4.57E-06 | 7.07E-01 | Positive |
| Role of NANOG in Mammalian Embryonic Stem Cell Pluripotency | 6.61E-06 | 1.34E+00 | Positive |
| RANK Signaling in Osteoclasts | 7.08E-06 | 2.45E+00 | Positive |
| ERK/MAPK Signaling | 1.00E-05 | 3.33E-01 | Positive |
| HGF Signaling | 1.12E-05 | 1.13E+00 | Positive |
| IL-17A Signaling in Fibroblasts | 1.29E-05 | - | - |
| IL-33 Signaling Pathway | 1.38E-05 | 7.07E-01 | Positive |
| WNT/Ca+ pathway | 1.41E-05 | 0.00E+00 | - |
| Tumor Microenvironment Pathway | 1.45E-05 | 1.67E+00 | Positive |
| CD40 Signaling | 1.51E-05 | 1.63E+00 | Positive |
| IL-12 Signaling and Production in Macrophages | 1.74E-05 | -6.32E-01 | Negative |
| Systemic Lupus Erythematosus In B Cell Signaling Pathway | 1.82E-05 | 1.60E+00 | Positive |
| PPARα/RXRα Activation | 1.82E-05 | 3.78E-01 | Positive |
| NER (Nucleotide Excision Repair, Enhanced Pathway) | 1.91E-05 | -1.13E+00 | Negative |
| April Mediated Signaling | 2.14E-05 | 1.34E+00 | Positive |
| Breast Cancer Regulation by Stathmin1 | 2.14E-05 | -1.50E+00 | Negative |
| IGF-1 Signaling | 2.29E-05 | 2.00E+00 | Positive |
| Growth Hormone Signaling | 2.34E-05 | 1.63E+00 | Positive |
| B Cell Activating Factor Signaling | 2.40E-05 | - | - |
| Cardiac Hypertrophy Signaling (Enhanced) | 2.63E-05 | 3.21E+00 | Positive |
| Regulation Of The Epithelial Mesenchymal Transition By Growth Factors Pathway | 2.63E-05 | 2.33E+00 | Positive |
| AMPK Signaling | 2.69E-05 | -3.78E-01 | Negative |
| Role of WNT/GSK-3β Signaling in the Pathogenesis of Influenza | 2.95E-05 | 1.00E+00 | Positive |
| ERK5 Signaling | 2.95E-05 | 0.00E+00 | - |
| Necroptosis Signaling Pathway | 3.47E-05 | 7.07E-01 | Positive |
| Toll-like Receptor Signaling | 3.72E-05 | 1.00E+00 | Positive |
| IL-22 Signaling | 3.80E-05 | 1.00E+00 | Positive |
| Renal Cell Carcinoma Signaling | 3.98E-05 | 2.00E+00 | Positive |
| Neuroinflammation Signaling Pathway | 4.07E-05 | 9.05E-01 | Positive |
| IL-17A Signaling in Gastric Cells | 5.25E-05 | - | - |
| Role of JAK1, JAK2 and TYK2 in Interferon Signaling | 5.25E-05 | - | - |
| p38 MAPK Signaling | 5.37E-05 | -8.16E-01 | Negative |
| Calcium Signaling | 5.50E-05 | -8.16E-01 | Negative |
| UVC-Induced MAPK Signaling | 5.62E-05 | 2.00E+00 | Positive |
| PEDF Signaling | 6.03E-05 | 2.24E+00 | Positive |
| UVB-Induced MAPK Signaling | 6.17E-05 | 2.00E+00 | Positive |
| LPS-stimulated MAPK Signaling | 6.46E-05 | 8.16E-01 | Positive |
| PDGF Signaling | 7.41E-05 | 1.63E+00 | Positive |
| Lymphotoxin β Receptor Signaling | 7.41E-05 | - | - |
| Endocannabinoid Developing Neuron Pathway | 7.76E-05 | -3.78E-01 | Negative |
| CSDE1 Signaling Pathway | 8.13E-05 | 4.47E-01 | Positive |
| EGF Signaling | 8.71E-05 | 1.34E+00 | Positive |
| Protein Kinase A Signaling | 8.71E-05 | -3.78E-01 | Negative |
| P2Y Purigenic Receptor Signaling Pathway | 9.77E-05 | 1.13E+00 | Positive |
| Gα12/13 Signaling | 1.05E-04 | 1.63E+00 | Positive |
| Role of JAK2 in Hormone-like Cytokine Signaling | 1.05E-04 | 4.47E-01 | Positive |
| TNFR2 Signaling | 1.07E-04 | 2.00E+00 | Positive |
| PCP (Planar Cell Polarity) Pathway | 1.23E-04 | 1.34E+00 | Positive |
| IL-1 Signaling | 1.29E-04 | 1.63E+00 | Positive |
| TGF-β Signaling | 1.29E-04 | 0.00E+00 | - |
| UVA-Induced MAPK Signaling | 1.35E-04 | - | - |
| 4-1BB Signaling in T Lymphocytes | 1.55E-04 | - | - |
| IL-9 Signaling | 1.74E-04 | 1.00E+00 | Positive |
| Pyridoxal 5'-phosphate Salvage Pathway | 1.78E-04 | 1.00E+00 | Positive |
| MIF-mediated Glucocorticoid Regulation | 1.95E-04 | 1.00E+00 | Positive |
| Corticotropin Releasing Hormone Signaling | 1.95E-04 | 0.00E+00 | - |
| PTEN Signaling | 2.29E-04 | 3.78E-01 | Positive |
| T Cell Receptor Signaling | 2.63E-04 | 1.51E+00 | Positive |
| Circadian Rhythm Signaling | 2.95E-04 | - | - |
| PI3K Signaling in B Lymphocytes | 3.16E-04 | 2.33E+00 | Positive |
| NRF2-mediated Oxidative Stress Response | 3.24E-04 | 0.00E+00 | - |
| Neuregulin Signaling | 3.39E-04 | 1.34E+00 | Positive |
| IL-13 Signaling Pathway | 3.39E-04 | 8.16E-01 | Positive |
| Mitochondrial Dysfunction | 3.55E-04 | 1.00E+00 | Positive |
| Oncostatin M Signaling | 3.89E-04 | 1.00E+00 | Positive |
| Androgen Signaling | 4.17E-04 | 8.16E-01 | Positive |
| Neurotrophin/TRK Signaling | 4.17E-04 | 4.47E-01 | Positive |
| Th1 and Th2 Activation Pathway | 4.17E-04 | - | - |
| Role of RIG1-like Receptors in Antiviral Innate Immunity | 4.27E-04 | - | - |
| Renin-Angiotensin Signaling | 4.47E-04 | 2.00E+00 | Positive |
| Xenobiotic Metabolism AHR Signaling Pathway | 4.47E-04 | 4.47E-01 | Positive |
| Pathogen Induced Cytokine Storm Signaling Pathway | 4.57E-04 | 1.27E+00 | Positive |
| G-Protein Coupled Receptor Signaling | 4.79E-04 | 7.75E-01 | Positive |
| Role of IL-17F in Allergic Inflammatory Airway Diseases | 5.13E-04 | 0.00E+00 | - |
| 14-3-3-mediated Signaling | 5.62E-04 | 4.47E-01 | Positive |
| NOD1/2 Signaling Pathway | 5.75E-04 | -3.78E-01 | Negative |
| PFKFB4 Signaling Pathway | 6.03E-04 | -1.00E+00 | Negative |
| Tight Junction Signaling | 6.17E-04 | - | - |
| IL-6 Signaling | 6.31E-04 | 2.45E+00 | Positive |
| TNFR1 Signaling | 7.08E-04 | 2.00E+00 | Positive |
| MicroRNA Biogenesis Signaling Pathway | 7.24E-04 | -3.78E-01 | Negative |
| BMP signaling pathway | 7.76E-04 | 4.47E-01 | Positive |
| B Cell Receptor Signaling | 8.32E-04 | 3.33E-01 | Positive |
| Production of Nitric Oxide and Reactive Oxygen Species in Macrophages | 8.51E-04 | 1.13E+00 | Positive |
| Apelin Endothelial Signaling Pathway | 9.33E-04 | 2.45E+00 | Positive |
| CD27 Signaling in Lymphocytes | 1.00E-03 | - | - |
| Salvage Pathways of Pyrimidine Ribonucleotides | 1.07E-03 | 1.00E+00 | Positive |
| FAK Signaling | 1.10E-03 | 1.50E+00 | Positive |
| Melanocyte Development and Pigmentation Signaling | 1.20E-03 | -4.47E-01 | Negative |
| Apoptosis Signaling | 1.41E-03 | 4.47E-01 | Positive |
| IL-8 Signaling | 1.45E-03 | 1.00E+00 | Positive |
| Relaxin Signaling | 1.45E-03 | 8.16E-01 | Positive |
| Insulin Secretion Signaling Pathway | 1.48E-03 | 0.00E+00 | - |
| Protein Ubiquitination Pathway | 1.51E-03 | - | - |
| Multiple Sclerosis Signaling Pathway | 1.59E-03 | -1.13E+00 | Negative |
| IL-2 Signaling | 1.59E-03 | - | - |
| Epithelial Adherens Junction Signaling | 1.70E-03 | 0.00E+00 | - |
| Oxytocin Signaling Pathway | 1.74E-03 | 1.41E+00 | Positive |
| HMGB1 Signaling | 1.86E-03 | 1.63E+00 | Positive |
| IL-17A Signaling in Airway Cells | 2.09E-03 | 1.00E+00 | Positive |
| Role of JAK1 and JAK3 in γc Cytokine Signaling | 2.09E-03 | - | - |
| Inhibition of Angiogenesis by TSP1 | 2.14E-03 | - | - |
| Th1 Pathway | 2.57E-03 | 1.34E+00 | Positive |
| T Cell Exhaustion Signaling Pathway | 2.69E-03 | -3.78E-01 | Negative |
| TREM1 Signaling | 2.75E-03 | 2.00E+00 | Positive |
| NGF Signaling | 2.82E-03 | 4.47E-01 | Positive |
| Interferon Signaling | 3.02E-03 | - | - |
| Angiopoietin Signaling | 3.31E-03 | 1.00E+00 | Positive |
| GDNF Family Ligand-Receptor Interactions | 3.31E-03 | 1.00E+00 | Positive |
| Role of MAPK Signaling in Inhibiting the Pathogenesis of Influenza | 3.31E-03 | 1.00E+00 | Positive |
| Synaptic Long Term Potentiation | 3.80E-03 | -4.47E-01 | Negative |
| Role of Hypercytokinemia/hyperchemokinemia in the Pathogenesis of Influenza | 3.98E-03 | 0.00E+00 | - |
| GNRH Signaling | 4.27E-03 | 0.00E+00 | - |
| Th2 Pathway | 4.47E-03 | 2.00E+00 | Positive |
| Immunogenic Cell Death Signaling Pathway | 4.79E-03 | 0.00E+00 | - |
| STAT3 Pathway | 4.79E-03 | - | - |
| FGF Signaling | 5.13E-03 | 0.00E+00 | - |
| Regulation of Cellular Mechanics by Calpain Protease | 5.62E-03 | - | - |
| Systemic Lupus Erythematosus In T Cell Signaling Pathway | 5.89E-03 | 1.00E+00 | Positive |
| Unfolded protein response | 5.89E-03 | - | - |
| Pyroptosis Signaling Pathway | 6.03E-03 | 2.00E+00 | Positive |
| IL-23 Signaling Pathway | 6.03E-03 | - | - |
| ERBB Signaling | 6.76E-03 | 1.00E+00 | Positive |
| NUR77 Signaling in T Lymphocytes | 7.24E-03 | 1.89E+00 | Positive |
| Type II Diabetes Mellitus Signaling | 7.59E-03 | 4.47E-01 | Positive |
| Amyloid Processing | 7.59E-03 | - | - |
| Role of NFAT in Cardiac Hypertrophy | 8.91E-03 | 0.00E+00 | - |
| Transcriptional Regulatory Network in Embryonic Stem Cells | 9.33E-03 | - | - |
| CNTF Signaling | 1.05E-02 | - | - |
| Role of IL-17A in Arthritis | 1.10E-02 | - | - |
| Inflammasome pathway | 1.12E-02 | - | - |
| Antioxidant Action of Vitamin C | 1.18E-02 | -2.00E+00 | Negative |
| Role of MAPK Signaling in Promoting the Pathogenesis of Influenza | 1.20E-02 | 1.00E+00 | Positive |
| Wound Healing Signaling Pathway | 1.29E-02 | 8.16E-01 | Positive |
| Xenobiotic Metabolism CAR Signaling Pathway | 1.29E-02 | -1.34E+00 | Negative |
| Induction of Apoptosis by HIV1 | 1.45E-02 | - | - |
| Role of PI3K/AKT Signaling in the Pathogenesis of Influenza | 1.45E-02 | - | - |
| IL-17 Signaling | 1.51E-02 | 1.34E+00 | Positive |
| ERBB4 Signaling | 1.62E-02 | - | - |
| Remodeling of Epithelial Adherens Junctions | 1.62E-02 | - | - |
| Cardiac Hypertrophy Signaling | 1.74E-02 | 4.47E-01 | Positive |
| Regulation of IL-2 Expression in Activated and Anergic T Lymphocytes | 1.95E-02 | 2.45E+00 | Positive |
| fMLP Signaling in Neutrophils | 2.04E-02 | 1.00E+00 | Positive |
| Antiproliferative Role of Somatostatin Receptor 2 | 2.19E-02 | - | - |
| Leptin Signaling in Obesity | 2.29E-02 | - | - |
| Xenobiotic Metabolism Signaling | 2.34E-02 | - | - |
| Maturity Onset Diabetes of Young (MODY) Signaling | 2.46E-02 | - | - |
| Chemokine Signaling | 2.46E-02 | - | - |
| White Adipose Tissue Browning Pathway | 2.51E-02 | 0.00E+00 | - |
| NF-κB Activation by Viruses | 2.51E-02 | - | - |
| TR/RXR Activation | 3.02E-02 | - | - |
| IL-4 Signaling | 3.09E-02 | 3.78E-01 | Positive |
| Oxidized GTP and dGTP Detoxification | 3.16E-02 | - | - |
| TEC Kinase Signaling | 3.24E-02 | 1.13E+00 | Positive |
| eNOS Signaling | 3.24E-02 | - | - |
| TWEAK Signaling | 3.39E-02 | - | - |
| Notch Signaling | 3.55E-02 | - | - |
| Dendritic Cell Maturation | 3.72E-02 | -3.78E-01 | Negative |
| Ceramide Signaling | 3.72E-02 | - | - |
| cAMP-mediated signaling | 3.98E-02 | 4.47E-01 | Positive |
| Death Receptor Signaling | 4.07E-02 | - | - |
| Inhibition of ARE-Mediated mRNA Degradation Pathway | 4.07E-02 | - | - |
| VEGF Signaling | 4.37E-02 | - | - |
| Gαq Signaling | 4.37E-02 | - | - |
| Neuropathic Pain Signaling In Dorsal Horn Neurons | 4.68E-02 | - | - |
| Aldosterone Signaling in Epithelial Cells | 4.68E-02 | - | - |
